# Effects of Double Diffusive Convection and Inclined Magnetic Field on the Peristaltic Flow of Fourth Grade Nanofluids in a Non-Uniform Channel

**DOI:** 10.3390/nano12173037

**Published:** 2022-09-01

**Authors:** Yasir Khan, Safia Akram, Alia Razia, Anwar Hussain, H. A. Alsulaimani

**Affiliations:** 1 Department of Mathematics, University of Hafr Al-Batin, Hafr Al-Batin 31991, Saudi Arabia; 2 Military College of Signals (MCS), National University of Sciences and Technology, Islamabad 44000, Pakistan; 3 Department of Mechanical Engineering, School of Mechanical and Manufacturing Engineering, National University of Sciences and Technology, Islamabad 44000, Pakistan

**Keywords:** thermal and concentration convection, nanofluids, peristaltic flow, non-uniform channel, inclined magnetic field, fourth grade fluid

## Abstract

This study explored the impact of double diffusive convection and inclined magnetic field in nanofluids on the peristaltic pumping of fourth grade fluid in non-uniform channels. Firstly, a brief mathematical model of fourth grade fluid along inclined magnetic fields and thermal and concentration convection in nanofluids was developed. A lubrication approach was used to simplify the highly non-linear partial differential equations. An analytical technique was then used to solve the highly non-linear differential equations. The exact solutions for the temperature, nanoparticle volume fraction and concentration were calculated. Numerical and graphical outcomes were also examined to see the effects of the different physical parameters of the flow quantities. It was noted that as the impact of Brownian motion increased, the density of the nanoparticles also increased, which led to an increase in the nanoparticle fraction. Additionally, it could be observed that as the effects of thermophoresis increased, the fluid viscosity decreased, which lowered the fraction of nanoparticles that was made up of less dense particles.

## 1. Introduction

The phenomenon of the peristaltic transport of fluid is an eminent topic within current research. It has gained popularity due to its practical contributions to the fields of biomechanics, engineering, technology and industry. It is a vital process in many physiological functions. The human body transports fluids from one organ to another using this procedure. For example, food movement through the oesophagus, fluid movement along the gastrointestinal tract, the vasomotor activity of veins, arteries and capillaries, the excretion of waste by the kidneys and other fluid movements are all conducted by peristaltic occurrences. Numerous studies have been carried out to explore peristaltic transport theoretically, experimentally and numerically. Latham was the first to incorporate viscous fluids in the peristalsis phenomenon, both analytically and experimentally [1]. Another classic work has formed the basis of this field, which uses the limitations of long wavelengths and small Reynolds numbers [2]. Non-Newtonian fluids have recently attracted a lot of attention in scientific research due to their use in various applications, including fabric glass production, starch suspensions, petroleum production, paper pulp production, polymer production, cement slurry production, the polymer processing industry and biological fluids. Non-Newtonian fluids are fluids that defy Newton’s law and change their viscosity in response to external stimuli. A few examples of non-Newtonian fluids are blood, shampoo, tomato ketchup, mud, honey, plastic, paint, pulp, polymer melts and concentrated juice. Non-Newtonian fluids can be divided into two categories: those whose shear stress is solely dependent on the shear rate and those whose shear rate and time characteristics include both elastic and viscous features. Since they are complicated fluids, they cannot be characterised by a single model. Therefore, numerous models have been used to describe the proposed behaviour of non-Newtonian fluids. Some other non-Newtonian fluids include Casson fluid, viscoelastic fluid, tangent hyperbolic fluid, second grade fluid and fourth grade fluid, which exhibit non-linear behaviour. Significant investigations can be found in the literature [3,4,5,6,7,8,9,10].

Choi [11] used the word “nanofluid” to describe a liquid that contains very small metallic or non-metallic particles of nanometre size and fibres, which are called nanoparticles. Masuda et al. [12] further explained the essential property of nanofluids, i.e., the amplification of thermal conductivity. This property enables the use of nanofluids in multiple engineering applications [13], mostly high-level nuclear systems. Das and Choi [14] reviewed the process of heat transfer. Similarly, Das et al. [15] further explained the mechanism of heat transfer in nanofluids. Moreover, Wang and Mujumdar [16] also studied the heat transfer properties of nanofluids, while Buongiorno [17] explained the absolute velocity of nanoparticles. He used the term “slip velocity” for the aggregate of the relative and base fluid velocity. He also based his study on the seven slip mechanisms: gravity settling, inertia, Brownian diffusion, Magnus effect, fluid drainage, thermophoresis and diffusiophoresis. He summed up his analysis with the conclusion that in the absence of tempestuous effects, thermophoresis and Brownian diffusion are significant. Based on these effects, he derived some conservation equations. The different flow geometries in nanofluids are the centre of attention in contemporary research. Nadeem and Akbar [18] examined the peristaltic flow of nanofluids in relation to endoscopic effects, which was the initial contribution to the peristaltic literature of nanofluids. Other works have since extended the recent research on the peristaltic flow of nanofluids [19,20,21,22,23,24,25].

Magnetohydrodynamics (MHD) is defined as a scientific field in which highly conductive fluid motion is studied in proximity to magnetic fields. The key aspect to be studied is the interplay between pressure increases and MHD. The MHD flow of nanofluids through channels via peristalsis is important, particularly with reference to certain problems that involve conductive physiological nanofluid movements. Such problems include the treatment of nuclear waste, the study of geothermal sources, the control of pollution that was caused by underground chemicals, the design of MHD power generators, the reduction in surgical blood loss, the treatment of tumours in stagnating hyperthermia, the selection of drug transfer utilising magnetic particles and blood pump machines and theoretical research on the operation of a peristaltic MHD compressor. Landeghem et al. [26] examined the magnetic nanoparticles that are induced in tumours and then heated up by alternating magnetic fields. Human tissues have very low magnetic susceptibility; therefore, the impacts of magnetic fields are not significant. However, there is a possibility that eddy currents could be produced in any biological tissue by electromagnetic fields [27,28]. Further research on the MHD peristaltic flow of nanofluids has also been carried out [29,30,31,32,33,34,35].

In the process of double diffusion, heat and mass transmissions take place simultaneously with the collusion of fluid motion. Double diffusion is indispensable in many disciplines, such as biology, solid state physics, geophysics, chemical engineering, astrophysics and oceanography [36]. Other related domains include engineering fields, such as crystal manufacturing, storage tanks for natural gas, solar ponds and the process of metal solidification. The double diffusive convection of peristaltic transport is a key area of attention for researchers nowadays. In [37], a model for peristaltic pumping with double diffusive convection in nanofluids was simulated using Mathematica software. Other authors have studied the effects of double diffusion on nanofluids using Newtonian base models. In [38,39,40,41,42,43,44,45], research on double diffusion was extended.

Limited work was found in our literature review on the impacts of inclined magnetic fields with double diffusive convection on peristaltic flow. Hence, this was considered in the current study using non-Newtonian fluids.

From the above discussion, the impacts of heat convection and magnetic flux on double diffusion convection cannot be neglected. The study of fourth grade fluids in the presence of double diffusion and inclined MHD has not yet been studied. This study theoretically expanded the previous research on the fluid models that are currently available. In the field of medical sciences, the flows of fluids with varying densities have practical significance. The analysis of flows in the human body during certain procedures, such as CT angiography, thallium stress testing and other procedures with a related topic, served as the inspiration for this study. Our first test involved injecting a dye, which was followed by an X-ray of the coronary arteries to check for blockages. In a subsequent test, a radioactive liquid called a radioisotope was injected into a human vein to gauge how well the blood flowed into the heart during exercise and rest. This study involved a mixture of areas of study, including biology, physics and material science (nanoscience).

In this study, the fundamental natural rules that regulate the operation of biological systems were described in mathematical terms. We selected a non-Newtonian model due to their widespread applications in technology. This work could aid in our theoretical comprehension of several biological flows. It could be beneficial for scientists and engineers who are engaged in the manufacture of CT and MRI devices, as well as other medical and biotechnological technologies. The rationale of this study was to show how magnetic fields and double diffusion convection affect peristaltic flow.

## 2. Mathematical Formulation

We considered the incompressible hydromagnetic flow of an electrically conductive fourth grade fluid in a non-uniform channel. The *x*-axis was drawn along the wave propagation and the *y*-axis was normal to it. We additionally regarded the magnetic fields as being slanted at an angle of ϕ. The lower wall of the channel was kept at a temperature of $T_{1}$, a solute concentration of $C_{1}$ and a nanoparticle concentration of $\Theta_{1}$, whereas the upper wall had a temperature of $T_{0},$ a solute concentration of $C_{0}$ and a nanoparticle concentration of $\Theta_{0}$.

**Figure 1:**
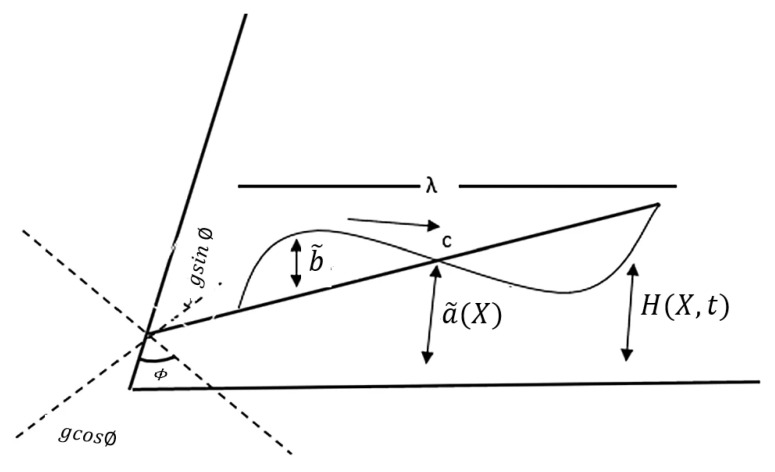
The geometry of the problem.

The geometrical shape of the surface wall is depicted in Figure 1 and is mathematically described in [5] as:

(1) $$H\left(X,t\right)=\tilde{a}\left(X\right)+\tilde{b}\sin\left(\frac{2\pi }{\lambda }\left(X-ct\right)\right),\;\;\;\;\;\;\;$$

where $\tilde{a}($*X*$)=b_{0}+b_{1}$ *X*, λ is the wavelength, $\tilde{a}$ denotes the channel half width at the axial distance *X*, $b_{0}$ is the half width at the inlet, $\left(b_{1}<<1\right)$ is a constant, $\tilde{b}$ represents the wave amplitude and c and t denote wave speed and time, respectively.

The stress tensor for fourth grade fluids was defined in [9] as:

(2) $$S=\mu {\tilde{A}}_{1}+{\tilde{\alpha }}_{1}{\tilde{A}}_{2}+{\tilde{\alpha }}_{2}{\tilde{A}}_{1}^{2}+{\tilde{\beta }}_{1}{\tilde{A}}_{3}+{\tilde{\beta }}_{2}\left({\tilde{A}}_{1}{\tilde{A}}_{2}+{\tilde{A}}_{2}{\tilde{A}}_{1}\right)+{\tilde{\beta }}_{3}\left(trac{\tilde{A}}_{1}^{2}\right){\tilde{A}}_{1}+{\tilde{\gamma }}_{1}{\tilde{A}}_{4}\;\;\;+{\tilde{\gamma }}_{2}\left({\tilde{A}}_{3}{\tilde{A}}_{1}+{\tilde{A}}_{1}{\tilde{A}}_{3}\right)+{\tilde{\gamma }}_{3}{\tilde{A}}_{2}^{2}+{\tilde{\gamma }}_{4}\left({\tilde{A}}_{1}^{2}{\tilde{A}}_{2}+{\tilde{A}}_{2}{\tilde{A}}_{1}^{2}\right)+{\tilde{\gamma }}_{5}trac\left({\tilde{A}}_{2}\right){\tilde{A}}_{2}+{\tilde{\gamma }}_{6}trac\left({\tilde{A}}_{2}\right){\tilde{A}}_{1}^{2}\;\;\;\;\;\;\;\;\;\;\;\;\;\;\;+\left({\tilde{\gamma }}_{7}trac{\tilde{A}}_{3}+{\tilde{\gamma }}_{8}trac{\tilde{A}}_{2}{\tilde{A}}_{1}\right){\tilde{A}}_{1},$$

(3) $${\tilde{A}}_{1}=\left(\nabla V\right)+{\left(\nabla V\right)}^{\tilde{T}},$$

(4) $${\tilde{A}}_{i}=\frac{d{\tilde{A}}_{i-1}}{dt}+{\tilde{A}}_{i-1}\left(\nabla V\right)+{\left(\nabla V\right)}^{\tilde{T}}{\tilde{A}}_{i-1},$$

where μ represents the constant viscosity, ${\tilde{\alpha }}_{1},$ ${\tilde{\alpha }}_{2},$ ${\tilde{\beta }}_{1}-{\tilde{\beta }}_{3}$ and ${\tilde{\gamma }}_{1}-{\tilde{\gamma }}_{8}$ stand for the material constants, $\tilde{T}$ represents the transpose and ${\tilde{A}}_{i}$ are Rivlin–Ericksen tensors.

The velocity field for a 2-dimensional and 2-directional flow was V=(U(X,Y,t),V(X,Y,t),0).

Within a laboratory framework (*X*, *Y*), the equations of motion for nanofluids and inclined magnetic fields for 2-dimensional incompressible flows were described in [37] as:

(5) $$\frac{\partial U}{\partial X}+\frac{\partial V}{\partial Y}=0,$$

(6) $$\rho_{f}\left(\frac{\partial }{\partial t}+U\frac{\partial }{\partial X}+V\frac{\partial }{\partial Y}\right)U=-\frac{\partial P}{\partial X}+\frac{\partial }{\partial X}\left(S_{XX}\right)+\frac{\partial }{\partial Y}\left(S_{XY}\right)-\sigma B_{0}^{2}\cos\phi \left(U\cos\phi -V\sin\phi \right)\;+g\{\left(1-\Theta_{0}\right)\rho_{f0}\left\{\beta_{T}\left(T-T_{0}\right)+\beta_{C}\left(C-C_{0}\right)\right\}-\left(\rho_{p}-\rho_{f0}\right)\left(\Theta -\Theta_{0}\right)\},$$

(7) $$\begin{array}{c} \rho_{f}\left(\frac{\partial }{\partial t}+U\frac{\partial }{\partial X}+V\frac{\partial }{\partial Y}\right)V=-\frac{\partial P}{\partial Y}+\frac{\partial }{\partial X}\left(S_{YX}\right)+\frac{\partial }{\partial Y}\left(S_{YY}\right)\\ +\sigma B_{0}^{2}\sin\phi \left(U\cos\phi -V\sin\phi \right), \end{array}$$

(8) $$\begin{array}{c} {\left(\rho c\right)}_{f}\left(\frac{\partial }{\partial t}+U\frac{\partial }{\partial X}+V\frac{\partial }{\partial Y}\right)T=\varepsilon \left(\frac{\partial^{2}T}{\partial X^{2}}+\frac{\partial^{2}T}{\partial Y^{2}}\right)+{\left(\rho c\right)}_{p}\{D_{B}\left(\frac{\partial \Theta }{\partial X}\frac{\partial T}{\partial X}+\frac{\partial \Theta }{\partial Y}\frac{\partial T}{\partial Y}\right)\\ \left(\frac{D_{T}}{T_{0}}\right)\left[{\left(\frac{\partial T}{\partial X}\right)}^{2}+{\left(\frac{\partial T}{\partial Y}\right)}^{2}\right]\}+D_{TC}\left(\frac{\partial^{2}C}{\partial X^{2}}+\frac{\partial^{2}C}{\partial Y^{2}}\right),\; \end{array}$$

(9) $$\left(\frac{\partial }{\partial t}+U\frac{\partial }{\partial X}+V\frac{\partial }{\partial Y}\right)C=D_{s}\left(\frac{\partial^{2}C}{\partial X^{2}}+\frac{\partial^{2}C}{\partial Y^{2}}\right)+D_{TC}\left(\frac{\partial^{2}T}{\partial X^{2}}+\frac{\partial^{2}T}{\partial Y^{2}}\right),\;\;\;\;\;$$

(10) $$\left(\frac{\partial }{\partial t}+U\frac{\partial }{\partial X}+V\frac{\partial }{\partial Y}\right)\Theta =D_{B}\left(\frac{\partial^{2}\Theta }{\partial X^{2}}+\frac{\partial^{2}\Theta }{\partial Y^{2}}\right)+\left(\frac{D_{T}}{T_{0}}\right)\left(\frac{\partial^{2}T}{\partial X^{2}}+\frac{\partial^{2}T}{\partial Y^{2}}\right),\;\;\;\;\;\;$$

It is known that flows are unsteady in a fixed frame (*X*, *Y*) but motion is steady in a wave frame (*x*, *y*), so the relationship between a fixed frame (*X*, *Y*) and wave frame was defined as:

(11) y=Y, x=X−ct, v=V, u=U−c, p(x,y)=P(X,Y,t).       

We then defined the following dimensionless quantities:

(12) y¯=yb0,x¯=xλ, v¯=vc,u¯=uc, δ=b0λ, p¯=b02pμcλ, t¯=ctλ, Re=ρfcb0μ,θ=T−T0T1−T0, h¯=hb0,  γ=C−C0C1−C0,Pr=(ρc)f υε, u=∂Ψ∂y, v=−δ∂Ψ∂x,Le=υDs, Ω=Θ−Θ0Θ1−Θ0,M=σμB0b0, NCT=DCT(T1−T0)(C1−C0)Ds,NTC=DCT(C1−C0)ς(T1−T0),Grt=gb02(1−Θ0)(T1−T0)ρfβTμ0c,Ln=υDB,Grc=g(1−Θ0)ρfβc(C1−C0)b02μ0c,Nb=(ρc)pDB(Θ1−Θ0)ς, Nt=(ρc)pDT(T1−T0)T0ς,GrF=g(ρp−ρf)(Θ1−Θ0)μ0cb02 , λ˜n=α˜ncμb0(n=1,2), ξ˜n=β˜nc2μb02(n=1,2,3),η˜n=γ˜nc3μb03(n=1−8),

In the above dimensionless quantities, g, $\rho_{f_{0}},$ $\rho_{p},$ δ, Pr, Re, $G_{rc},$ $G_{rT},$ $G_{rF},$ Le, Ln, $N_{b},$ $N_{t},$ M, $N_{CT},$ $N_{TC},$ θ, Ω, $\gamma ,\;\beta_{C},$ $\beta_{T},$ ε, ${\left(\rho c\right)}_{p}$ and ${\left(\rho c\right)}_{f}$ represent acceleration due to gravity, the density of the fluid at $T_{0}$, the density of the particles, wave number, Prandtl number, Reynolds number, solute Grashof number, thermal Grashof number, nanoparticle Grashof number, Lewis number, nanofluid Lewis number, the Brownian motion parameter, thermophoresis parameter, Hartmann number, Soret parameter, Dufour parameter, dimensionless temperature, solute (species) concentration, nanoparticle fraction, the volumetric solute expansion coefficient of the fluid, the volumetric thermal expansion coefficient of a fluid, thermal conductivity, nanoparticle heat capacity and fluid heat capacity, respectively.

Equation (10) in dimensionless form became:

(13) h=1+mx+βsin(2πx),    

where $\beta =\frac{b}{b_{0}}$ is the amplitude ratio or occlusion and $m=\frac{b_{1}}{b_{0}}.$

By means of Equations (11) and (12), Equation (5) was automatically satisfied and Equations (6)–(10) were transformed for stream function Ψ, temperature θ, nanoparticle fraction γ and solute concentration Ω in a wave frame (after dropping bars):

(14) Reδ(ΨyΨxy−ΨxΨyy)=−∂p∂x+δ∂Sxx∂x+∂Sxy∂y−M2cosϕ((Ψy+1)cosϕ+Ψxδsinϕ) +Grtθ+Grcγ−GrFΩ,      

(15) Reδ3(ΨxΨxy−ΨyΨxx)=−∂p∂y+δ2∂Syx∂x+δ∂Syy∂y+M2δsinϕ((Ψy+1)cosϕ+Ψxδsinϕ),

(16) RePrδ(Ψyθx−Ψxθy)=(θyy+δ2θxx)+NTC(δ2γxx+γyy)+Nb(δ2Ωxθx+θyΩy) +Nt(δ2(θx)2+(θy)2),    

(17) ReδLe(Ψyγx−Ψxγy)=(δ2γxx+γyy)+NCT(δ2θxx+θyy),   

(18) ReδLn(ΨyΩx−ΨxΩy)=(δ2Ωxx+Ωyy)+NtNb(δ2θxx+θyy),     

Then, by employing the presumption of long wavelengths and low Reynolds numbers, Equations (14)–(18) became:

(19) $$0=-\frac{\partial p}{\partial x}+\frac{\partial S_{xy}}{\partial y}-M^{2}\cos^{2}\phi \left(\Psi_{y}+1\right)+G_{rt}\theta +G_{rc}\gamma -G_{rF}\Omega ,\;\;\;\;$$

(20) $$0=-\frac{\partial p}{\partial y},\;\;$$

(21) $$\frac{\partial^{2}\theta }{\partial y^{2}}+N_{TC}\frac{\partial^{2}\gamma }{\partial y^{2}}+N_{b}\left(\frac{\partial \theta }{\partial y}\frac{\partial \Omega }{\partial y}\right)+N_{t}{\left(\frac{\partial \theta }{\partial y}\right)}^{2}=0,\;\;$$

(22) $$\frac{\partial^{2}\gamma }{\partial y^{2}}+N_{CT}\frac{\partial^{2}\theta }{\partial y^{2}}=0,\;\;$$

(23) $$\frac{\partial^{2}\Omega }{\partial y^{2}}+\frac{N_{t}}{N_{b}}\frac{\partial^{2}\theta }{\partial y^{2}}=0,\;\;\;\;$$

By eliminating the pressure from Equations (19) and (20), we yielded:

(24) $$\frac{\partial^{2}S_{xy}}{\partial y^{2}}-M^{2}\cos^{2}\phi \frac{\partial^{2}\Psi }{\partial y^{2}}+G_{rt}\frac{\partial \theta }{\partial y}+G_{rc}\frac{\partial \gamma }{\partial y}-G_{rF}\frac{\partial \Omega }{\partial y}=0,\;\;$$

where:

(25) $$S_{xy}=\frac{\partial^{2}\Psi }{\partial y^{2}}+2\Gamma {\left(\frac{\partial^{2}\Psi }{\partial y^{2}}\right)}^{3},\;\;\;\;\;\;\;\;\;\;\;\;\;$$

and $\Gamma ={\tilde{\xi }}_{2}+{\tilde{\xi }}_{3}$ stands for the Deborah number.

The boundary conditions in the wave frame that related to the stream function Ψ, temperature θ, nanoparticle fraction Ω and solute concentration γ were defined as follows [5]:

(26) $$\begin{array}{c} \Psi =0,\;\frac{\partial^{2}\Psi }{\partial y^{2}}=0\;\mathrm{on}\;y=0,\\ \Psi =F,\;\frac{\partial \Psi }{\partial y}=-1\mathrm{on}\;y=h\left(x\right)=1+mx+\beta \sin\left(2\pi x\right), \end{array}$$

(27) θ=0 on y=0 and θ=1 on y=h(x),

(28) Ω=0 on y=0 and Ω=1 on y=h(x),

(29) γ=0 on y=0 and γ=1 on y=h(x),

where F is the mean flow rate in the wave frame (dimensionless form), which could be related to the mean flow Q using Q=F+1 and $F=\int_{0}^{h}\frac{\partial \Psi }{\partial y}\cdot dy\;$.

## 3. Different Wave Forms

The expressions for the considered wave forms (in dimensionless form) were defined as follows [5]:

(1) Multi-sinusoidal wave:

h(x)=1+mx+βsin(2lπx)

(2) Trapezoidal wave:

$$h\left(x\right)=1+mx+\beta \left(\frac{32}{\pi^{2}}\sum_{l=1}^{\infty }\frac{\;\sin\left(2\pi \left(2l-1\right)x\right)}{{\left(2l-1\right)}^{2}}\sin\left(\frac{\pi }{8}\left(2l-1\right)\right)\right),$$

(3) Triangular wave:

$$h\left(x\right)=1+mx+\beta \left(\frac{8}{\pi^{3}}\sum_{l=1}^{\infty }\frac{{\left(-1\right)}^{l+1}\sin\left(2\pi \left(2l-1\right)x\right)}{{\left(2l-1\right)}^{2}}\right)$$

(4) Square wave:

$$h\left(x\right)=1+mx+\beta \left(\frac{4}{\pi }\sum_{l=1}^{\infty }\frac{{\left(-1\right)}^{l+1}\cos\left(2\left(2l-1\right)\pi x\right)}{\left(2l-1\right)}\right)$$

## 4. Solution to the Problem

### 4.1. Exact Solution

The exact solution for the nanoparticle volume fraction that satisfied Boundary Condition (28) was defined as:

(30) $$\Omega =\frac{N_{t}\left(e^{-\omega y}-1\right)}{N_{b}\left(1-e^{-h\omega }\right)}+\frac{y}{h}\left(\frac{N_{t}}{N_{b}}+1\right),$$

The exact solution for the solute (species) concentration that satisfied Boundary Condition (29) was defined as:

(31) $$\gamma =\frac{N_{\mathrm{CT}}\left(e^{-\omega y}-1\right)}{1-e^{-h\omega }}+\frac{y\left(1+N_{\mathrm{CT}}\right)}{h},$$

The exact solution for the temperature that satisfied Boundary Condition (27) was defined as:

(32) $$\theta =\frac{e^{-\omega y}-1}{e^{-h\omega }-1},$$

where:

(33) $$\omega =\frac{N_{b}+N_{t}}{h\left(1-N_{\mathrm{CT}}N_{\mathrm{TC}}\right)},$$

### 4.2. Numerical Solution

Since Equations (24) and (19) were non-linear differential equations, calculating exact solutions for these equations was difficult. The non-linear equations were illustrated utilising ND Solve in Mathematica computational software. Thus, graphical illustrations were created for the numerical approximations of the solutions.

Special Cases:

- The results of this study were reduced to the classical results of viscous fluids when $\Gamma =M=G_{rt}=G_{rc}\gamma =G_{rF}=m=0$. This special case corresponded to a very simplified model in a comparison to the present model since it only related to Newtonian viscous flows in the absence of nanofluid and thermophysical phenomena.
- The results of Bég and Tripathi [37] could be used for our problem in the limited case when Γ=M=m=0.

## 5. Graphical Outcomes

**Figure 2:**
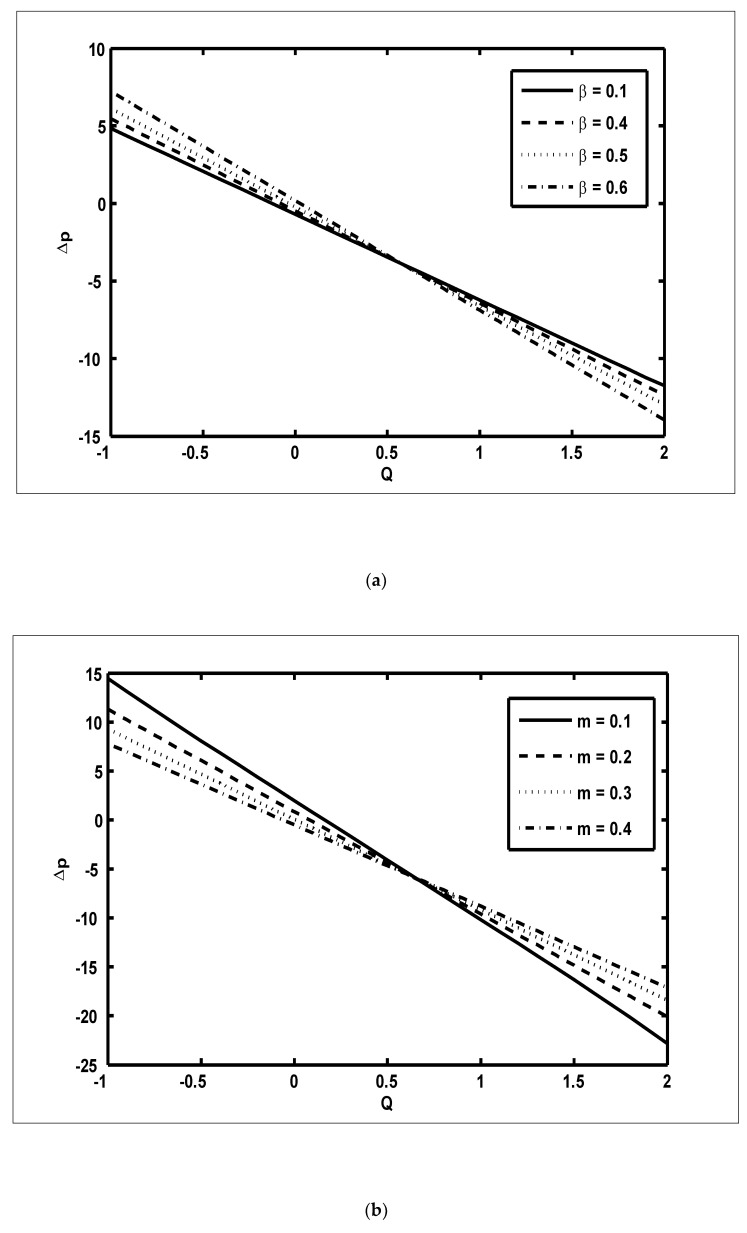
The pressure increase over one wavelength (Δp) against the volume flow rate (*Q*): (**a**) $N_{CT}=0.3,\;N_{TC}=0.7,\;G_{rc}=0.4,\;G_{rF}=0.1,\;G_{rt}=0.5,\;N_{t}=0.7,\;N_{b}=0.2,\;M=4,\;\phi =\frac{\pi }{6},\;\Gamma =0.8,\;m=0.4$; (**b**) $N_{CT}=0.3,\;N_{TC}=0.7,\;G_{rc}=0.4,\;G_{rF}=0.1,\;G_{rt}=0.5,\;N_{t}=0.7,\;N_{b}=0.2,\;M=4,\;\phi =\frac{\pi }{6},\;\Gamma =0.8,\;\beta =0.6$.

**Figure 3:**
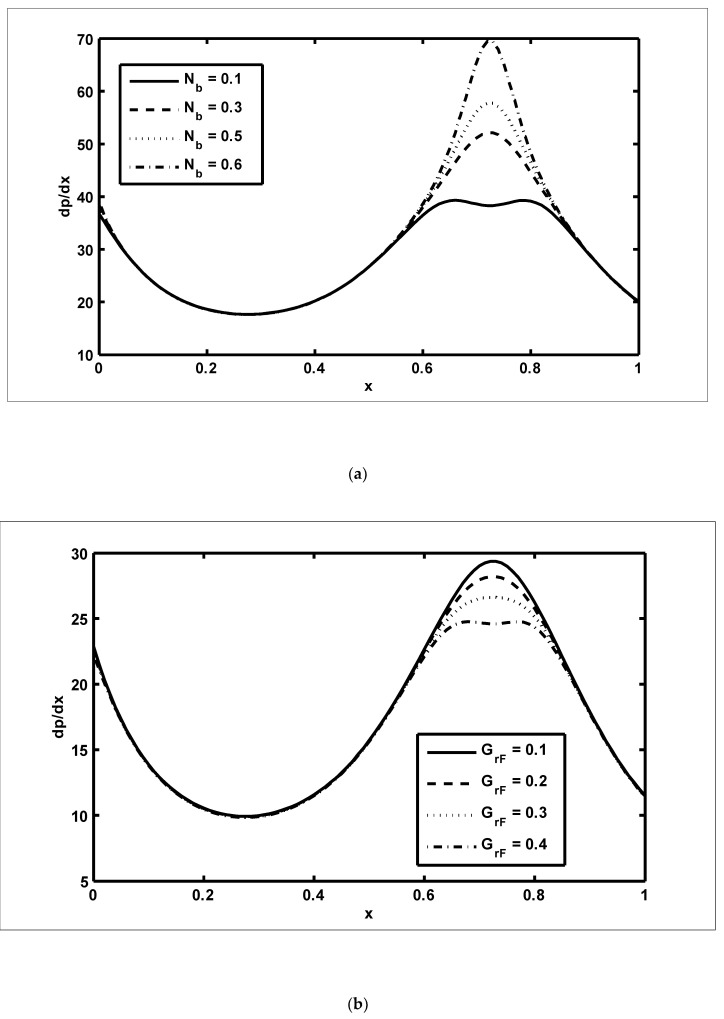
The pressure gradient (dp/dx) against the axial distance (x): (**a**) $N_{CT}=0.3,\;N_{TC}=0.7,\;G_{rc}=0.4,\;G_{rF}=0.1,\;G_{rt}=0.5,\;N_{t}=0.7,\;M=4,\;\phi =\frac{\pi }{6},\;\Gamma =0.8,\;\beta =0.4,\;m=0.4,\;Q=-5$; (**b**)$N_{CT}=0.3,\;N_{TC}=0.7,\;G_{rc}=0.4,\;N_{b}=0.1,\;G_{rt}=0.5,\;N_{t}=0.7,\;M=4,\;\phi =\frac{\pi }{6},\;\Gamma =0.8,\;\beta =0.4,\;m=0.4,\;Q=-5$.

**Figure 4:**
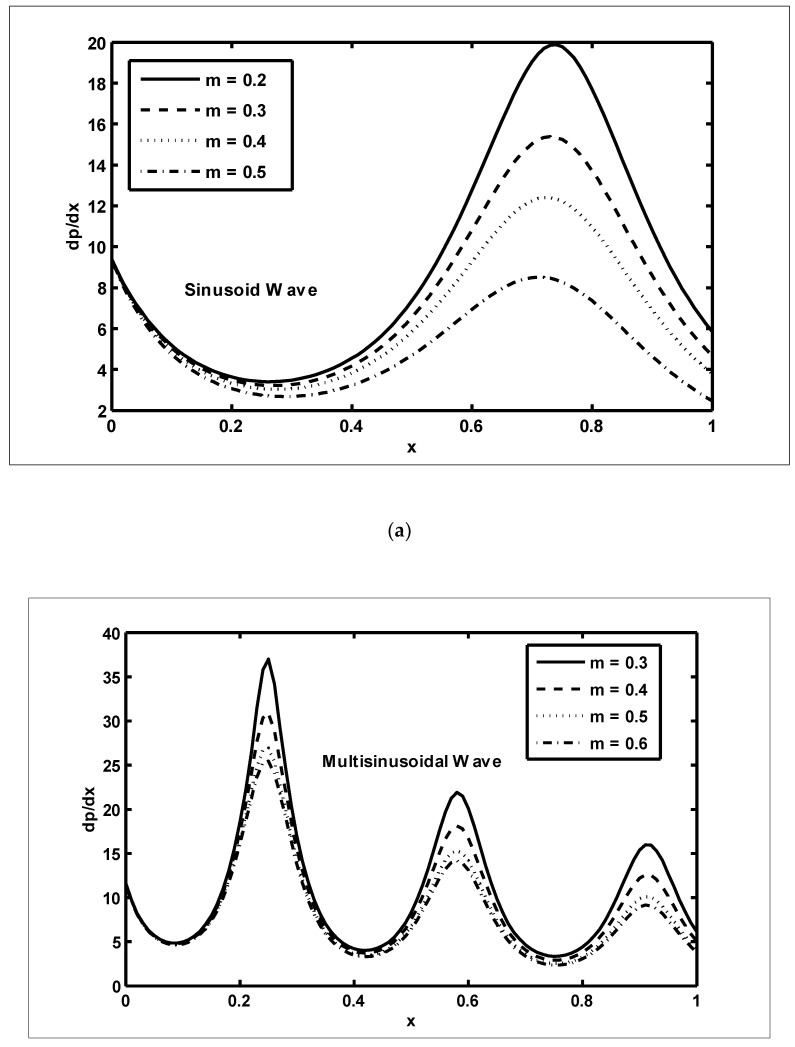

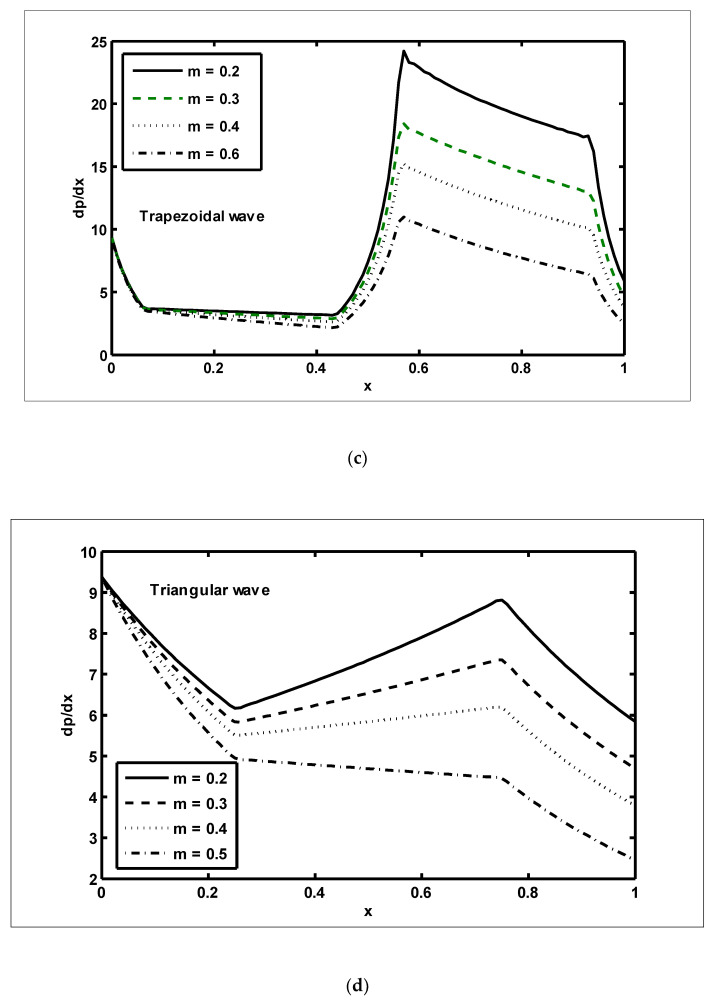
The pressure gradient (dp/dx) against the axial distance (x) for the different wave shapes: The various parameters used in (**a**–**d**) are as: $N_{CT}=0.3,\;N_{TC}=0.7,\;G_{rc}=0.4,\;G_{rF}=0.1,\;G_{rt}=0.5,\;N_{t}=0.7,\;N_{b}=0.9,M=4,\;\phi =\frac{\pi }{6},\;\Gamma =0.8,\;\beta =0.4,\;m=0.4,\;Q=-5\;$

In this section, we present the graphical outcomes of the problem under consideration. To observe the effects of pressure increase with volume flow rate Q, Figure 2a,b was plotted for diverse values of β and m. To analyse the impact of the pressure increase, the zones of peristaltic flow were spilt into the following regions: (a) the retrograde (Δp>0,Q<0) zone, in which the flow was travelling in the opposite direction from the peristaltic motion; (b) the peristaltic (Δp>0,Q>0) zone, in which positive values of Q were completely due to peristalsis after responding to the difference in pressure; (c) the free pumping (Δp=0) zone; and (d) the co-pumping (Δp<0,Q>0) region, in which the difference in pressure supported the flow because of the surface walls. From Figure 2a, it can be seen that in the retrograde, peristaltic and free pumping regions, the pressure increased due to the increasing values of β, whereas in the co-pumping region, the pressure decreased due to the increasing values of β. It is obvious from Figure 2b that pressure decreased with the increasing values of m in the retrograde and peristaltic pumping regions but this behaviour was reversed in the co-pumping region. To reveal the impact of pressure gradient on $N_{b}$ and $G_{rF}$, Figure 3a,b were plotted. It can be seen in Figure 3a that when x∈[0.6,0.9], the pressure gradient increased due to the increasing values of $N_{b}.$ Figure 3b was plotted to detect the impact of the pressure gradient for various values of $G_{rF}.$ It can be seen in this figure that when x∈[0.6,0.9], the pressure gradient decreased with the increasing values of $G_{rF}.$ In order to examine the consequences of different wave forms on the pressure gradient, Figure 4a–d were plotted. It can be seen in these figures that the maximum pressure gradient was observed in the trapezoidal wave.

**Figure 5:**
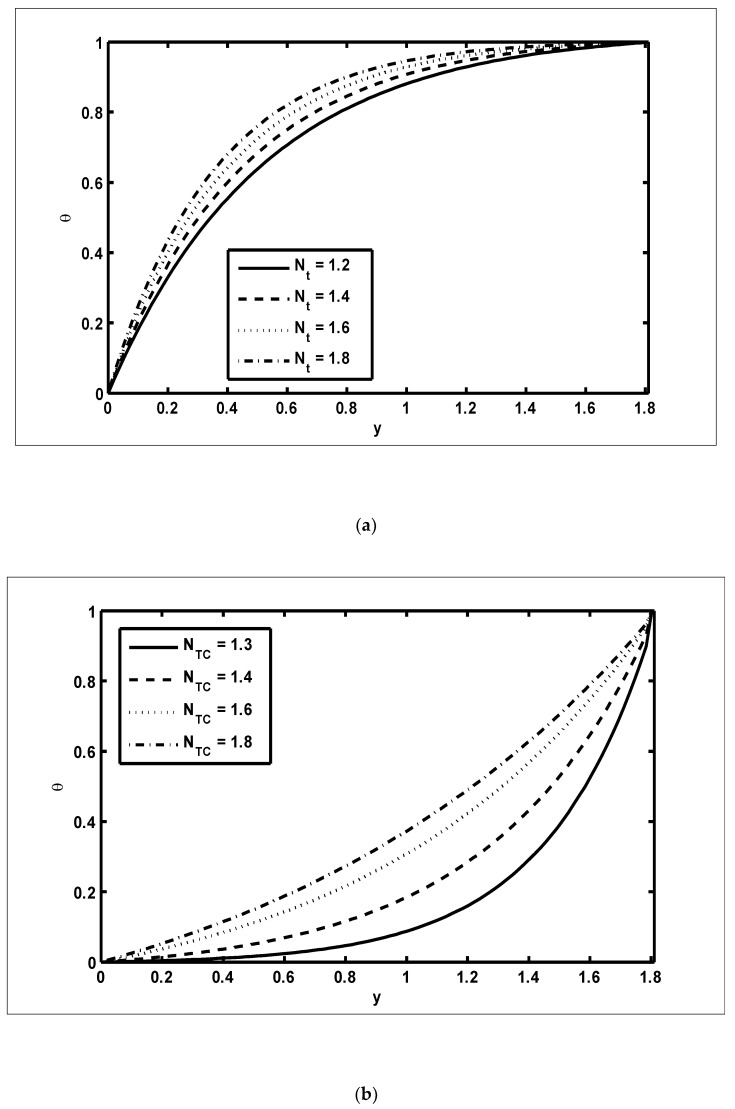
The temperature profiles (*θ*): (**a**) $N_{CT}=0.9,\;N_{TC}=0.7,\;N_{b}=0.9,\;\beta =0.4,\;m=0.2,\;x=0.2$; (**b**) $N_{CT}=0.9,\;N_{t}=0.7,\;N_{b}=0.9,\;\beta =0.4,\;m=0.2,\;x=0.2$.

**Figure 6:**
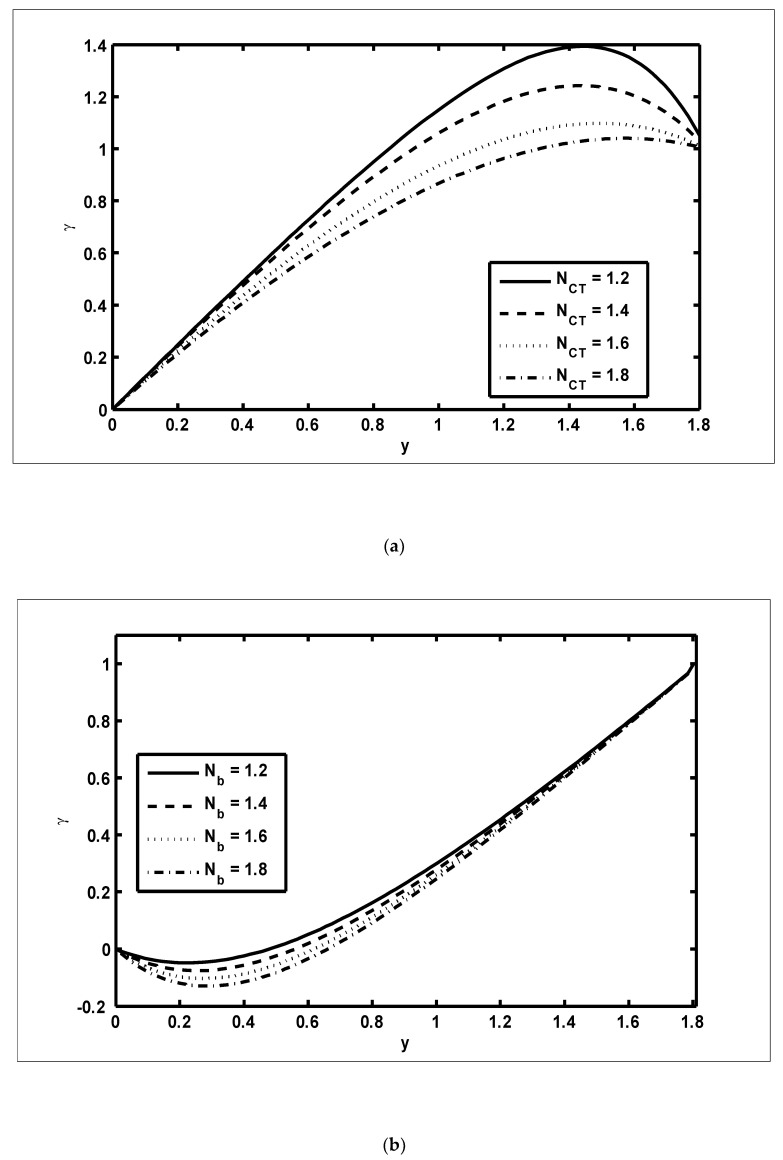
The solute concentration profiles (γ): (**a**) $N_{CT}=0.9,\;N_{TC}=0.7,\;N_{t}=0.9,\;\beta =0.4,\;m=0.2,\;x=0.2$; (**b**) $N_{CT}=0.9,\;N_{b}=0.7,\;N_{t}=0.9,\;\beta =0.4,\;m=0.2\;,x=0.2$.

**Figure 7:**
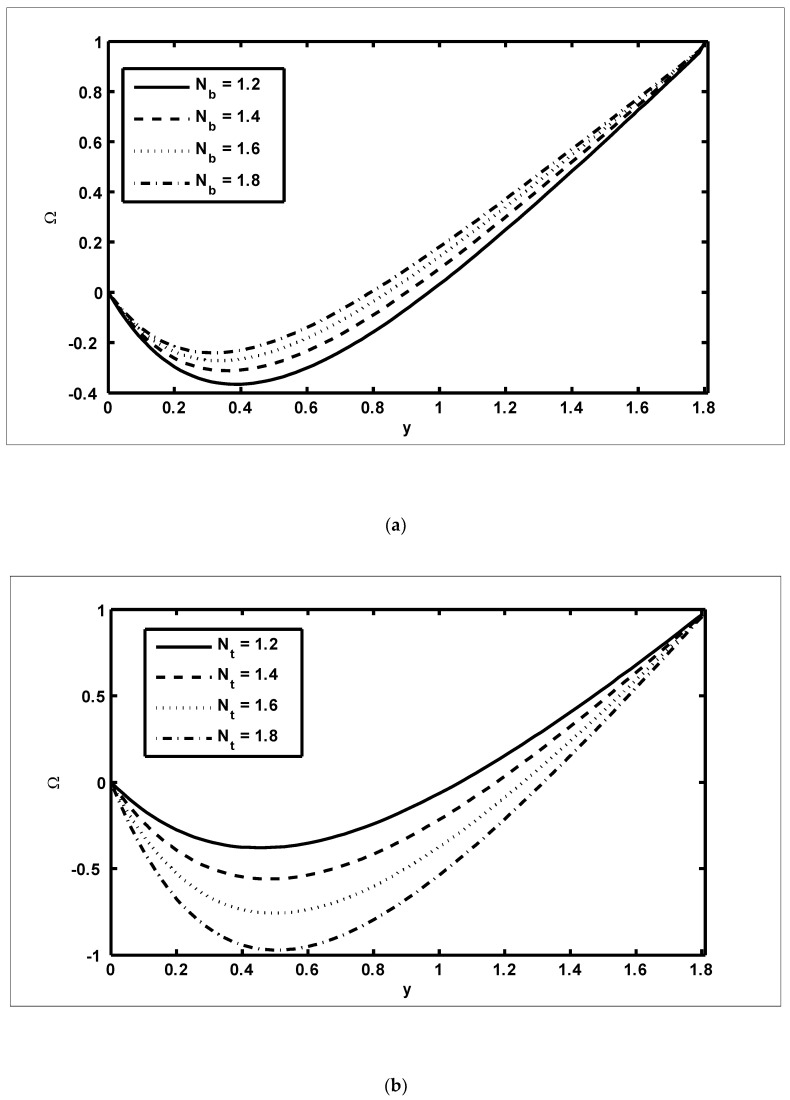
The nanoparticle fraction profiles (Ω): (**a**) $N_{CT}=0.9,\;N_{TC}=0.7,\;N_{t}=0.9,\;\beta =0.4,\;m=0.2,\;x=0.2$; (**b**) $N_{CT}=0.9,\;N_{TC}=0.7,N_{b}=0.9,\;\beta =0.4,\;m=0.2,\;x=0.2$.

To study the features of the temperature profiles, solute concentrations and nanoparticle fractions, Figure 5, Figure 6 and Figure 7 were plotted. The difference between the temperature of hot gas and cold surfaces produced a valuable source known as thermophoresis. Additionally, this caused the particles to move in the direction of the cold surfaces. It should be noted that in this study, the heat transfer changed as the thermophoresis $N_{t}$ parameter varied. Figure 5a illustrates that the temperature profiles increased because of the increasing values of $N_{t}.$ Similar effects can be noted in Figure 5b for the case of the Dufour $N_{TC}$ parameter. Physically, it was evident that the Dufour effect, which is also known as the diffusion-thermo effect, characterised the heat flow that was produced whenever the chemical system was subjected to a concentration gradient. It can be seen in Figure 6a,b that the solute concentration profiles decreased because of the increasing values of $N_{b}$ and $N_{CT}.$ This behaviour was because $N_{b}$ and $N_{CT}$ had a direct relationship with each other. Moreover, it occurred when random motions interacted with of solid nanoparticles through random collisions and micro-mixing, which dispersed the solid nanoparticles and lowered their concentration. Figure 7a,b were drawn to observe the effects of the nanoparticle fractions on $N_{b}$ and $N_{t}$. Increasing values of $N_{b}$ increased the nanoparticle density, which caused the nanoparticle fraction to grow (Figure 7a), whereas the nanoparticle fraction decreased with an increase in $N_{t}$ Figure 7b. When $N_{t}$ increased, the fluid viscosity lessened, which resulted in a reduction in the nanoparticle fraction of less dense particles.

**Figure 8:**
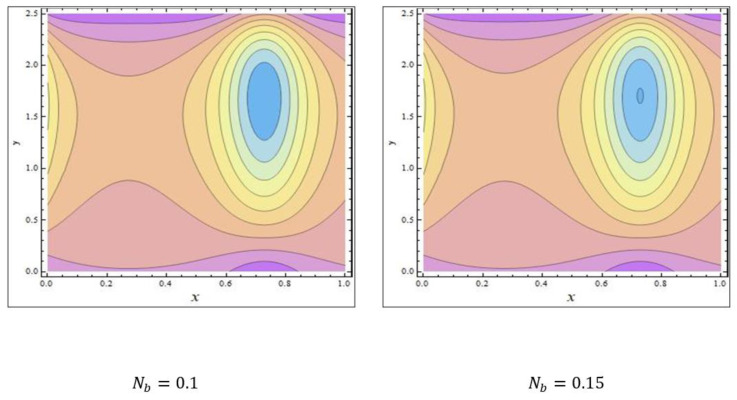
The streamlines of $N_{b}$. The values of the other parameters were $N_{CT}=0.3,\;N_{TC}=0.7,\;G_{rc}=0.4,\;G_{rF}=0.1,\;G_{rt}=0.5,\;N_{t}=0.7,\;M=3.5,\;\phi =\frac{\pi }{6},\Gamma =0.8,\;\beta =0.7,\;m=0.6,\;Q=2.37$.

**Figure 9:**
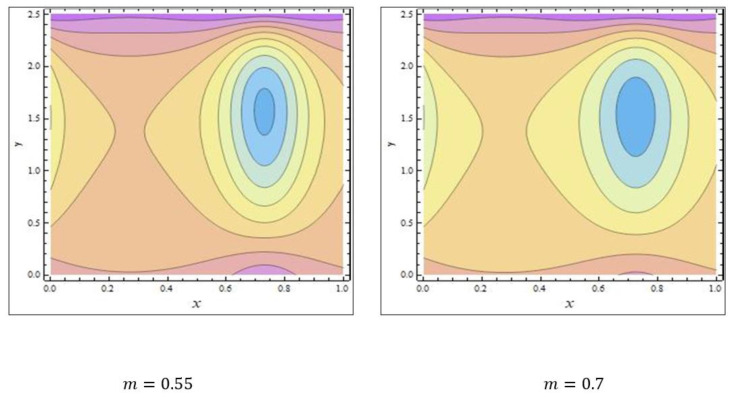
The streamlines of m. The values of the other parameters were $N_{CT}=0.3,\;N_{TC}=0.7,\;G_{rc}=0.4,\;G_{rF}=0.1,\;G_{rt}=0.5,\;N_{t}=0.7,\;M=3.5,\;\phi =\frac{\pi }{6},\;\Gamma =0.8,\;\beta =0.7,\;Q=2.37$.

**Figure 10:**
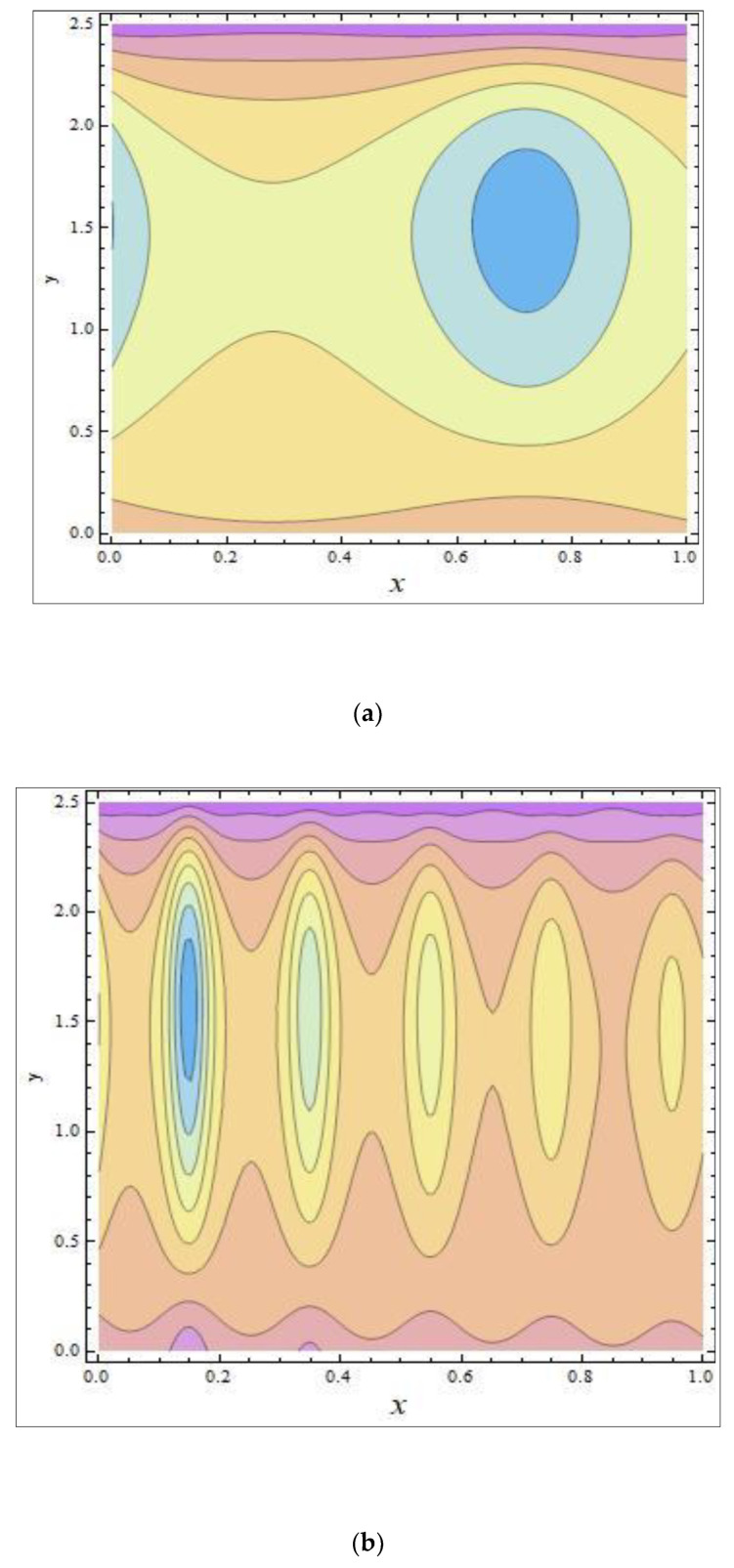

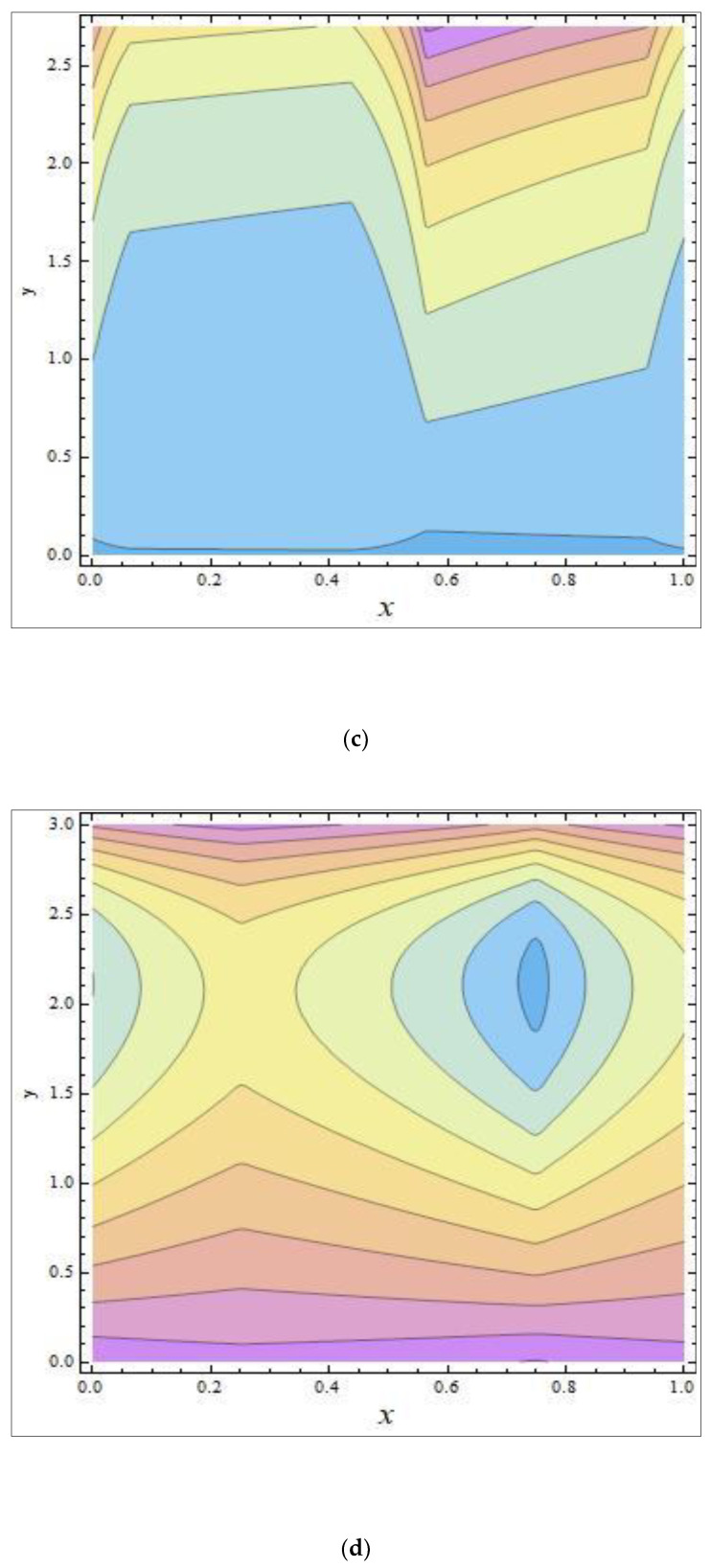
The streamlines of different wave shapes: (**a**) sinusoidal wave; (**b**) multi-sinusoidal wave; (**c**) trapezoidal wave; (**d**) triangular wave. The values of the other parameters were $N_{CT}=0.3,\;N_{TC}=0.7,\;G_{rc}=0.4,\;G_{rF}=0.1,\;G_{rt}=0.5,\;N_{t}=0.7,\;M=3.5,\;\phi =\frac{\pi }{6},\;\Gamma =0.8,\;\beta =0.7,\;m=0.6,\;Q=2.37$.

In peristaltic propulsive flows, trapping is a rare phenomenon. It starts with the formation of a fluid mass that moves internally and is encircled by peristaltic wave streamlines. Using peristaltic waves with high rates of flow and substantial occlusions, streamlines catch the mass of fluid and propel it along. To study the phenomenon of trapping, Figure 8, Figure 9 and Figure 10 were plotted. The streamlines for the discrete values of $N_{b}$ are represented in Figure 8, which shows that with the enhanced values of $N_{b}$, the number and size of the trapped masses increased, while they decreased with increasing values of m (Figure 9). The streamlines for the different wave forms are shown in Figure 10. A comparison between this work and others in the available literature is presented in Table 1. It was noted that our results agreed with the results for viscous fluid and those of Bég and Tripathi [37].

## 6. Conclusions

This article explored the impacts of double diffusive convection and inclined magnetic fields in nanofluids on the peristaltic pumping of fourth grade fluids in non-uniform channels. A mathematical model of a fourth grade fluid with inclined magnetic fields and thermal and concentration convection in nanofluids was developed. A numerical technique was used to solve the highly non-linear differential equations. The exact solutions for the temperature, nanoparticle volume fraction and solute concentration were calculated. Graphical outcomes were also illustrated to observe the effects of the different physical parameters of the flow quantities. The main findings were as follows:

- The pressure gradient increased with increasing values of the Brownian motion parameter, whereas it decreased with increasing values of the nanoparticle Grashof number;
- The temperature profiles increased with increasing values of the thermophoresis parameter and Dufour parameter, while the concentration profiles decreased with increasing values of the Brownian motion parameter and Soret parameter;
- The nanoparticle fractions decreased with increasing values of the Brownian motion parameter, whereas they increased with increasing values of the thermophoresis parameter;
- The number and size of trapped masses increased with increasing values of the Brownian motion parameter, while they decreased with increasing values of the non-uniform parameter.

## Figures and Tables

**Table 1:** A comparison between the present work and the available literature.

	Velocity Profile (u)		
y=h(x)	Present Work	Viscous Fluid	Bég and Tripathi [37]
0	2.7954	2.89268	2.95852
0.119994	2.76701	2.85902	2.92114
0.239987	2.68047	2.75708	2.80836
0.359981	2.53245	2.58482	2.61918
0.479974	2.31785	2.33935	2.35242
0.599968	2.0296	2.01684	2.00664
0.719961	1.65823	1.61226	1.58005
0.839955	1.19132	1.1191	1.07026
0.959948	0.612152	0.528699	0.473961
1.07994	−0.103582	0.171164	−0.213634
1.19994	−1	−1	−1

## Data Availability

Not Applicable.

## References

[1] Latham T.W. (1966). Fluid Motion in a Peristaltic Pump. Master’s Thesis.

[2] Shapiro A.H., Jaffrin M.Y., Weinberg S.L. (1969). Peristaltic pumping with long wavelengths at low reynolds number. J. Fluid Mech..

[3] Haider S., Ijaz N., Zeeshan A., Li Y.-Z. (2019). Magneto-hydrodynamics of a solid-liquid two-phase fluid in rotating channel due to peristaltic wavy movement. Int. J. Numer. Methods Heat Fluid Flow.

[4] Riaz A., Ellahi R., Bhatti M.M., Marin M. (2019). Study of heat and mass transfer in the Eyring-Powell model of fluid propagating peristaltically through a rectangular complaint channel. Heat Transf. Res..

[5] Saleem N., Hayat T., Alsaedi A. (2012). Effects of induced magnetic field and slip condition on peristaltic transport with heat and mass transfer in a non-uniform channel. Int. J. Phys. Sci..

[6] Bhatti M.M., Zeeshan A., Tripathi D., Ellahi R. (2018). Thermally developed peristaltic propulsion of magnetic solid particles in Biorheological fluids. Indian J. Phys..

[7] Safia Akram K.S., Mekheimer Y., Elmaboud A. (2018). Particulate suspension slip flow induced by peristaltic waves in a rectangular duct: Effect of lateral walls. Alex. Eng. J..

[8] Akram S., Nadeem S. (2013). Influence of induced magnetic field and heat transfer on the peristaltic motion of a Jeffrey fluid in an asymmetric channel: Closed form solutions. J. Magn. Magn. Mater..

[9] Kothandapani M., Pushparaj V., Prakash J. (2018). Effect of magnetic field on peristaltic flow of a fourth grade fluid in a tapered asymmetric channel. J. King Saud Univ. Eng. Sci..

[10] Ijaz N., Riaz A., Zeeshan A., Ellahi R., Sait S.M. (2020). Buoyancy Driven Flow with Gas-Liquid Coatings of Peristaltic Bubbly Flow in Elastic Walls. Coatings.

[11] Choi S.U.S. (1995). Enhancing thermal conductivity of fluid with nanoparticles developments and Applications of non-Newtonian Flow. ASME J. Heat Transf..

[12] Masuda H., Ebata A., Teramae K., Hishinuma N. (1993). Alteration of thermal conductivity and viscosity of liquid by dispersing ultra-fine particles. Netsu Bussei.

[13] Buongiorno J., Hu W. Nanofluid coolants for advanced nuclear power plants. Proceedings of the ICAPP ’05.

[14] Das S.K., Choi S.U.S., Patel H.E. (2006). Heat transfer in nanofluids—A review. Heat Transf. Eng..

[15] Das S.K., Choi S.U., Yu W., Pradeep T. (2007). Nanofluid Science and Technology.

[16] Wang X.-Q., Mujumdar A.S. (2007). Heat transfer characteristics of nanofluids: A review. Int. J. Therm. Sci..

[17] Buongiorno J. (2006). Convective transport in nanofluids. J. Heat Transf..

[18] Akbar N.S., Nadeem S. (2011). Endoscopic effects on peristaltic flow of a nanofluid. Commun. Theor. Phys..

[19] Ellahi R., Zeeshan A., Hussain F., Asadollahi A. (2019). Peristaltic blood flow of couple stress fluid suspended with nanoparticles under the influence of chemical reaction and activation energy. Symmetry.

[20] Riaz A., Khan S.U.-D., Zeeshan A., Khan S.U., Hassan M., Muhammad T. (2021). Thermal analysis of peristaltic flow of nanosized particles within a curved channel with second-order partial slip and porous medium. J. Therm. Anal..

[21] Nadeem S., Riaz A., Ellahi R., Akbar N.S. (2013). Mathematical model for the peristaltic flow of Jeffrey fluid with nanoparticles phenomenon through a rectangular duct. Appl. Nanosci..

[22] Mekheimer K., Hasona W., Abo-Elkhair R., Zaher A. (2018). Peristaltic blood flow with gold nanoparticles as a third grade nanofluid in catheter: Application of cancer therapy. Phys. Lett. A.

[23] Ellahi R., Raza M., Akbar N.S. (2017). Study of peristaltic flow of nanofluid with entropy generation in a porous medium. J. Porous Media.

[24] Hussain F., Ellahi R., Zeeshan A., Vafai K. (2018). Modelling study on heated couple stress fluid peristaltically conveying gold nanoparticle through coaxial tube: A remedy for gland tumor and arthritis. J. Mol. Liq..

[25] Ramesh K., Prakash J. (2019). Thermal analysis for heat transfer enhancement in electroosmosis-modulated peristaltic transport of Sutterby nanofluids in a microfluidic vessel. J. Therm. Anal. Calorim..

[26] Landeghem F., Maier-Hauff K., Jordan A. (2009). Post mortem studies in glioblastoma patients treated with thermotherapy using magnetic nanoparticles. Biomaterials.

[27] Foster K. (2000). Thermal and nonthermal mechanisms of interaction of radio-frequency energy with biological systems. IEEE Trans. Plasma Sci..

[28] Zhu L., Xu L., Chencinski N. (1998). Quantification of the 3-D electromagnetic power absorption rate in tissue during transurethral prostatic microwave thermotherapy using heat transfer model. IEEE Trans. Biomed. Eng..

[29] Akram S. (2016). Nanofluid effects on peristaltic transport of a fourth grade fluid in the occurrence of inclined magnetic field. Sci. Iran..

[30] Kothandapani M., Prakash J. (2015). Effects of thermal radiation parameter and magnetic field on the peristaltic motion of Williamson nanofluids in a tapered asymmetric channel. Int. J. Heat Mass Transf..

[31] Habib D., Salamat N., Ahsan M., Abdal S., Siddique I., Ali B. (2022). Significance of bioconvection and mass transpiration for MHD micropolar Maxwell nanofluid flow over an extending sheet*. Waves Random Complex Media.

[32] Prakash J., Siva E.P., Tripathi D., Beg O.A. (2019). Thermal slip and radiative heat transfer effects on electroosmotic magneto nanoliquid peristaltic propulsion through a microchannel. Heat Transf. Asian Res..

[33] Awan A.U., Ahammad N.A., Majeed S., Gamaoun F., Ali B. (2022). Significance of hybrid nanoparticles, Lorentz and Coriolis forces on the dynamics of water based flow. Int. Commun. Heat Mass Transf..

[34] Wang F., Asjad M.I., Rehman S.U., Ali B., Hussain S., Gia T.N., Muhammad T., Williamson M.H.D. (2021). Nanofluid Flow over a Slender Elastic Sheet of Irregular Thickness in the Presence of Bioconvection. Nanomaterials.

[35] Khan S.A., Eze C., Lau K.T., Ali B., Ahmad S., Ni S., Zhao J. (2022). Study on the novel suppression of heat transfer deterioration of supercritical water flowing in vertical tube through the suspension of alumina nanoparticles. Int. Commun. Heat Mass Transf..

[36] Singh O.P., Srinivasan J. (2014). Effect of Rayleigh numbers on the evolution of double-diffusive salt fingers. Phys. Fluids.

[37] Bég O.A., Tripathi D. (2012). Mathematica simulation of peristaltic pumping with double-diffusive convection in nanofluids a bio-nanoengineering model, Proceedings of the Institution of Mechanical Engineers, Part N. J. Nanoeng. Nanosyst..

[38] Alolaiyan H., Riaz A., Razaq A., Saleem N., Zeeshan A., Bhatti M.M. (2020). Effects of double diffusion convection on Third grade nanofluid through a curved compliant peristaltic channel. Coatings.

[39] Akram S., Zafar M., Nadeem S. (2018). Peristaltic transport of a Jeffrey fluid with double-diffusive convection in nanofluids in the presence of inclined magnetic field. Int. J. Geom. Methods Mod. Phys..

[40] Kuznetsov A.V., Nield D.A. (2011). Double-diffusive natural convective boundary-layer flow of a nanofluid past a vertical plate. Int. J. Therm. Sci..

[41] Sharma A., Tripathi D., Sharma R.K., Tiwari A.K. (2019). Analysis of double diffusive convection in electroosmosis regulated peristaltic transport of nanofluids. Physica A.

[42] Nield D.A., Kuznetsov A.V. (2011). The onset of double diffusive convection in a nanofluid layer. Int. J. Heat Fluid Flow.

[43] Akram S., Razia A., Afzal F. (2020). Effects of velocity second slip model and induced magnetic field on peristaltic transport of non-Newtonian fluid in the presence of double-diffusivity convection in nanofluids. Arch. Appl. Mech..

[44] Asha S.K., Sunitha G. (2020). Thermal radiation and hall effects on peristaltic blood flow with double diffusion in the presence of nanoparticles. Case Stud. Therm. Eng..

[45] Narayana M., Sibanda P. (2012). On the solution of double-diffusive convective flow due to a cone by a linearization method. J. Appl. Math..

